# State Registration and Its Values

**Published:** 1920-09-25

**Authors:** 


### THE MATRONS' AND SISTERS' DEPARTMENT.

### STATE REGISTRATION AND ITS VALUES.

### V. How the College Achieved Registration.

Few nurses have any perception of the extent to which the founding of the College of Nursing changed the entire horizon of the nursing world. The only link which formerly bound nurses to their professional interests lay in the training-school. It is true that, leaving their *alma mater* to join some other institution, they might transfer their interests to their new field of work. But it remained true that all they knew about the profession they had entered, its ideals and opportunities, had been learned within the hospital walls. Outside, in private nursing, in district work, in nursing homes, and the like, it was the exception to find any incentive or common centre. Unless some communication were kept up with the matrons and sisters who first impressed the seal of their vocation on her mind, the nurse *qua* nurse was the most solitary individual in the world. If she were happy enough to belong to one of the Leagues or Guilds which unite former members of a school in close association she might enjoy some community of interests, but hardly in any other way.

The founding of the College gathered the scattered units together and gave them at once a professional rallying-point. It is difficult to realise the immediate effect of holding meetings to consider the future of the profession in every training-school all over the kingdom. And this is what followed the first founding of the College. For the first time a common impulse stirred in the minds of widely separated members of the profession, and a new life began to spring up vigorously in regions which previously seemed barren.

It is important to realise that in making itself felt through the medium of the training-schools the College took the only course which could unite the nursing world. The hospitals are the centre of nursing existence. Handed down from one generation to another in the person of the matrons and sisters, the ideals of Florence Nightingale are cherished, taught, and realised in the schools where the women who will form the next generation of nurses are moulded for their high duties. It is impossible to enter a good training-school, and they are far more numerous than the critics would have us believe, without appreciating the all-pervading presence of a great inspiration. This is what draws nurses together. In their daily work and study they become insensibly responsive to the call. Here are the roots from which all that is ripest and best in the nursing world is to grow. Here, if anywhere, the unity of the profession will see its birth.

It was to the training-schools, large and small, general and special, voluntary and municipal, that the promoters of the College appealed to founa a great central organisation to protect the interests of all. And the immediate effect was to send an electric current of interest and hope right through the nursing world. In every town and in many remote districts nurses were called together, and (in many instances for the first time in their lives) heard about the needs of the profession and the benefits which would accrue to it from the universal registration of trained nurses at the hands of the State.

All this happened some four years ago, and already the total of 20,000 members of the College which some daring spirits ventured to suggest at the beginning has nearly been reached. No longer is the cult of nursing ideals confined to the training-schools. There is now a local centre of the College, meaning professional life, instruction, debate, and interest, within reach, by a short railway journey of every member. But the training-schools still remain the strength of the College and its natural recruiting field.

It cannot be too clearly understood that the State registration of nurses was not only impossible but undesirable until nurses in overwhelming numbers had made it evident that they desired it. So long as the cry for State registration was heard faintly, from a small section of nurses out of sympathy with the rest, it could not move the Government to undertake this measure. Nothing but the united voice of the profession would justify the State in setting up machinery for its regulation of a far-reaching nature, such, as we have shown, might easily have done more harm than good in a disunited body. The College, having united the training-schools, had united the entire profession; the time for registration had come. Now in due course it is here.

The need for instruction about professional interests was never more insistent than at the present time. In every training-school, in every centre of the College, there is a call for courageous leading, in order that the women who are just entering upon their career, and those no less who have grown perhaps a little weary in it, should realise their responsibilities in this the greatest crisis the nursing world has hitherto experienced.

